# Supplementary figures and images for: Reduced Glucose Sensation Can Increase the Fitness of Saccharomyces cerevisiae Lacking Mitochondrial DNA
Source: PLoS One. 2016 Jan 11;11(1):e0146511. doi: 10.1371/journal.pone.0146511 (PMC4709096; doi:10.1371/journal.pone.0146511)

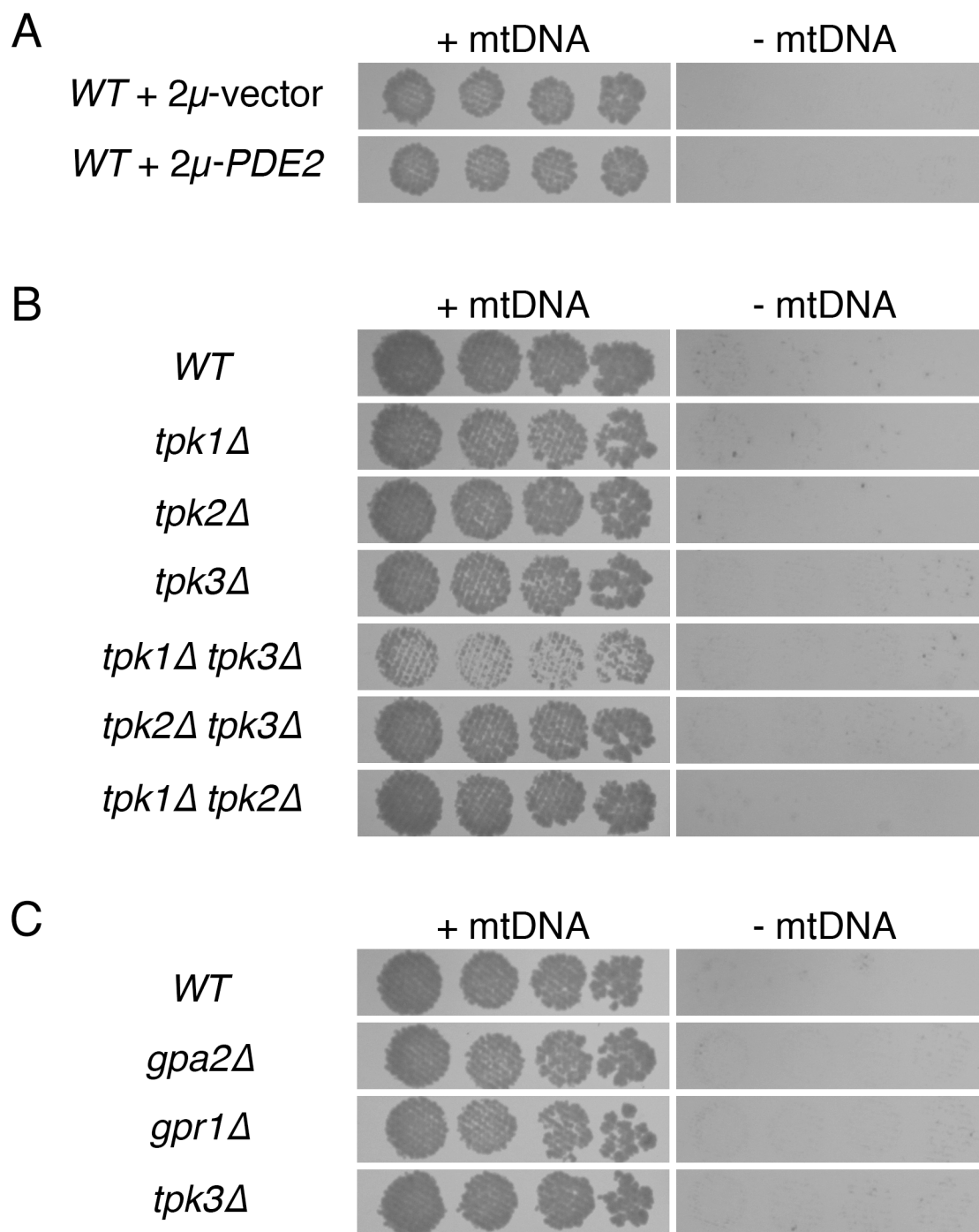

Supplement: S1 Fig — (A) Spot-dilution tests from Fig 1A were replica-plated to YPALac medium and incubated for 2 d. (B) Spot-dilution tests from Fig 1B were treated as in (A). (C) Spot-dilution tests from Fig 1C were treated as in (A). (PDF) [file pone.0146511.s001.pdf]

A

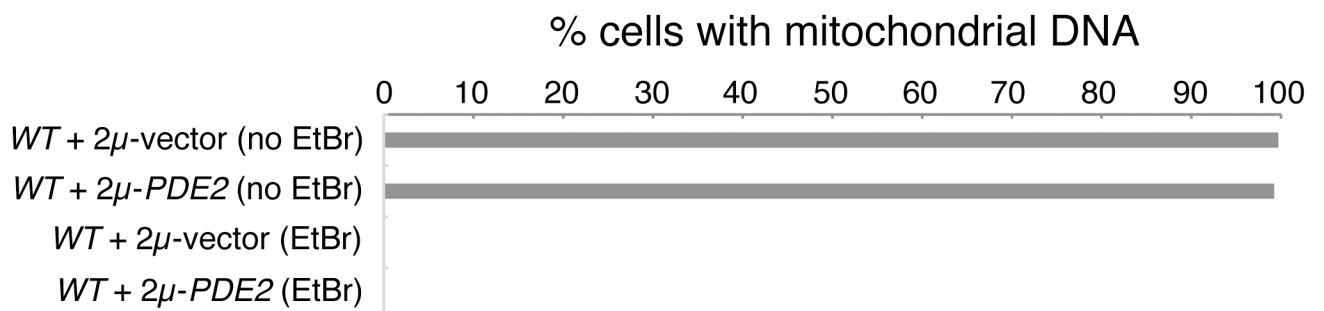

B

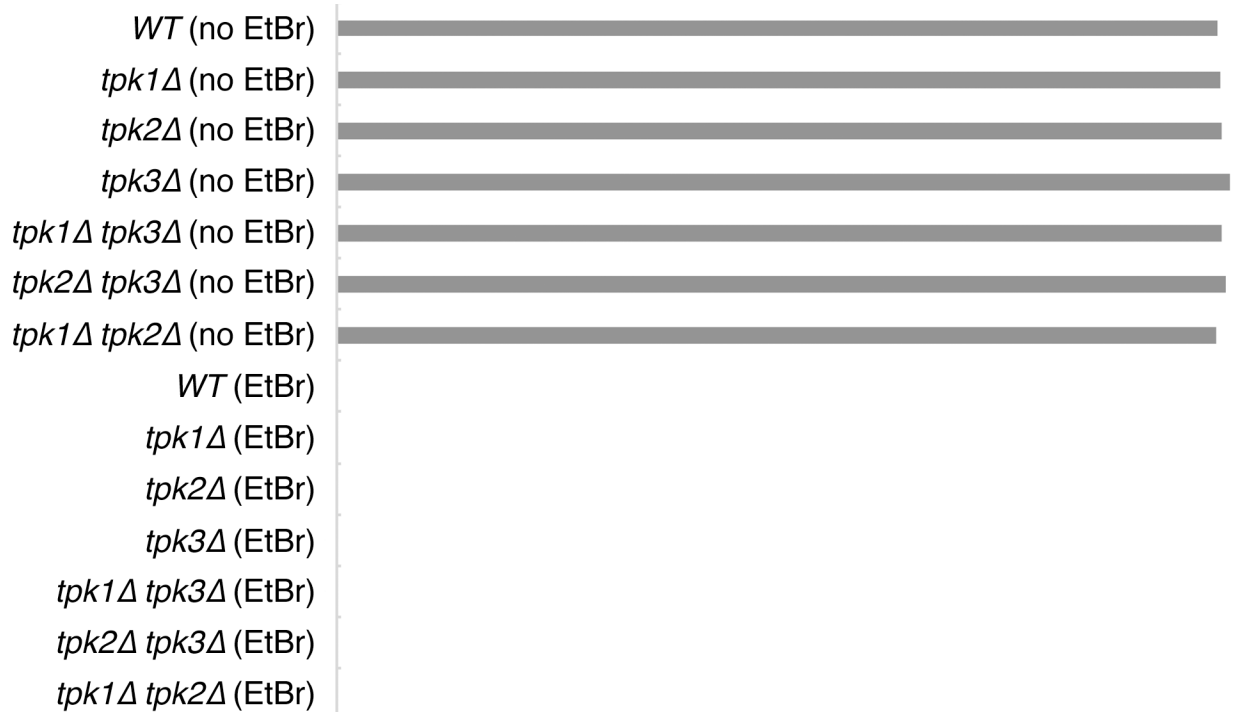

C

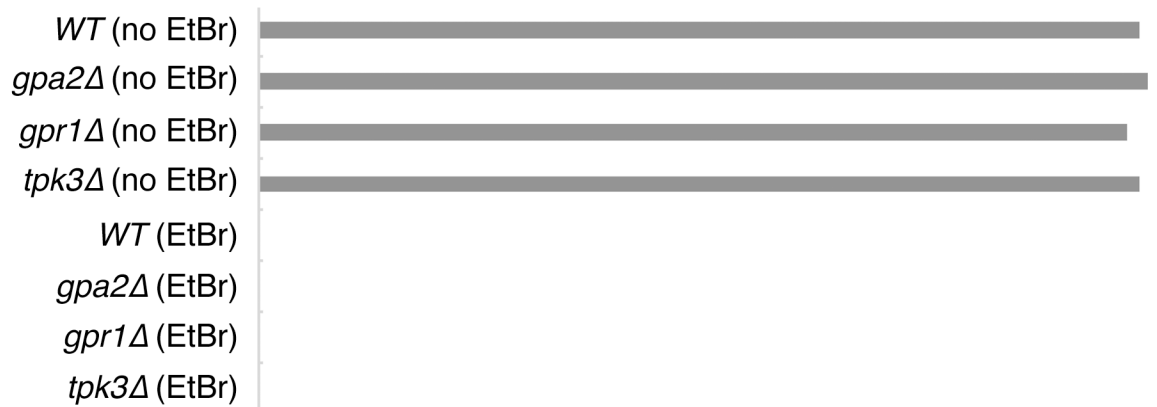

D

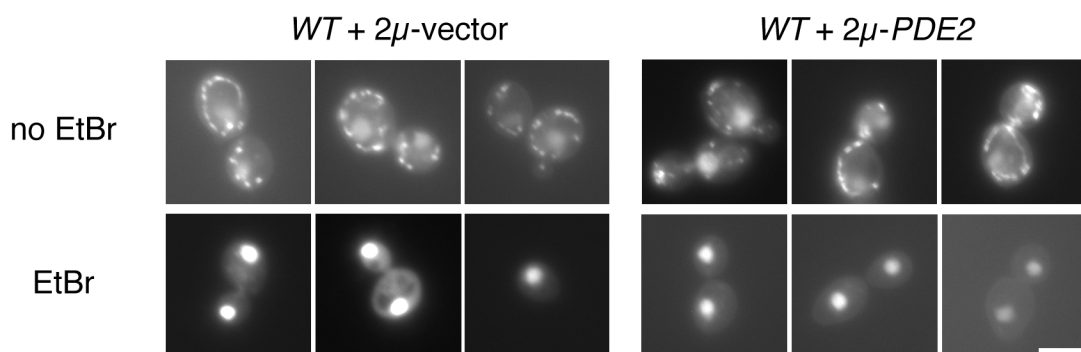

Supplement: S2 Fig — Cells were harvested from spot dilution test plates and cultured in liquid medium before DAPI staining (n > 200 cells for each strain under each condition). (A) Spot-dilution tests from Fig 1A were tested for the presence of mtDNA nucleoids. (B) Spot-dilution tests from Fig 1B were treated as in (A). (C) Spot-dilution tests from Fig 1C were treated as in (A). (D) Example images of cells assessed in (A). Scale bar, 5 μm. (PDF) [file pone.0146511.s002.pdf]

A

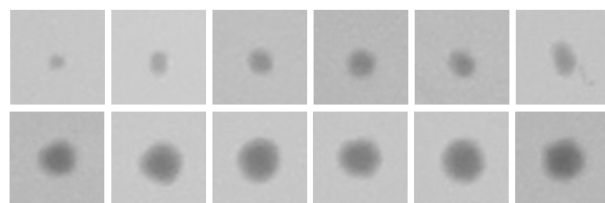*mip1Δ + 2μ-vector**mip1Δ + 2μ-PDE2*

B

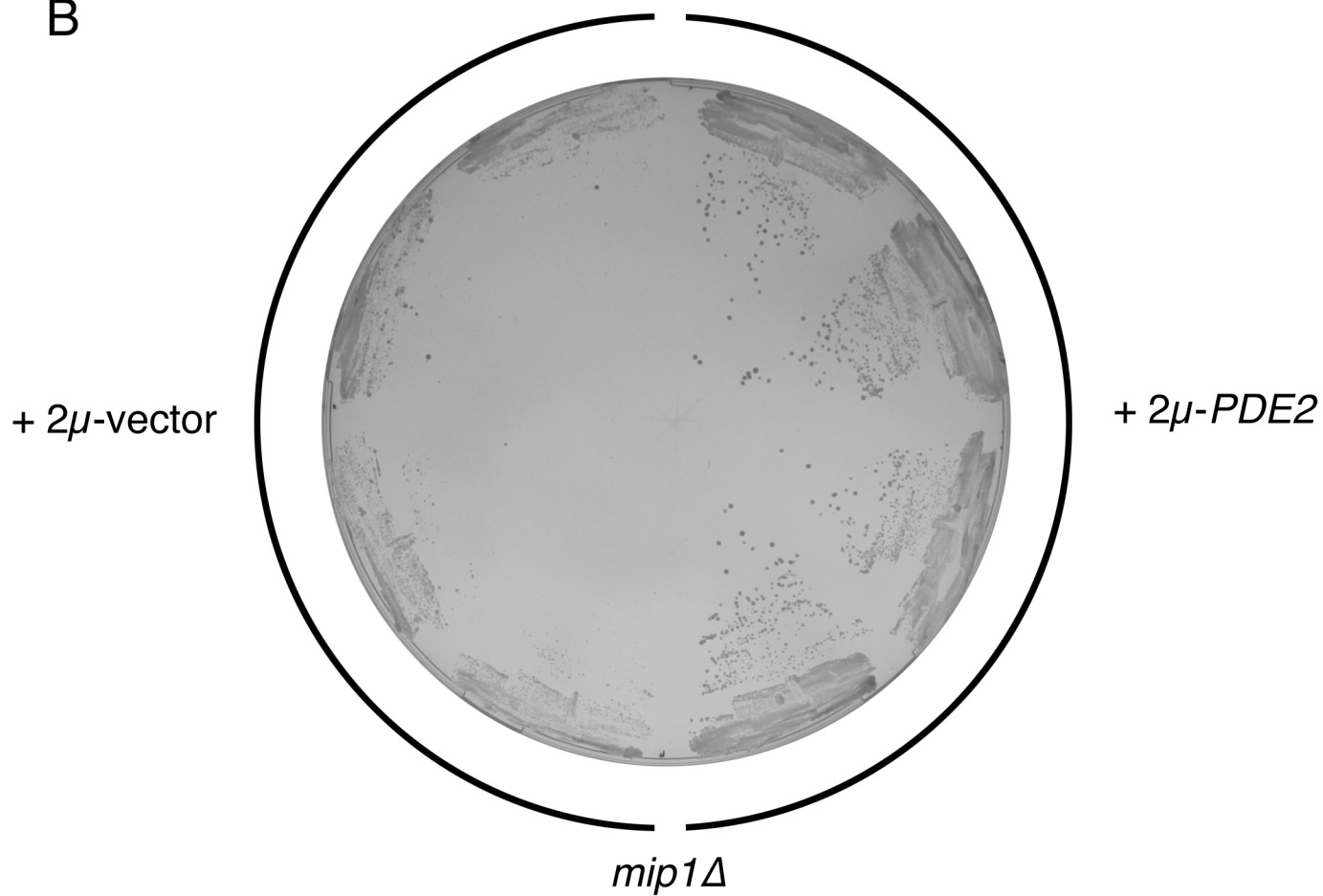

C

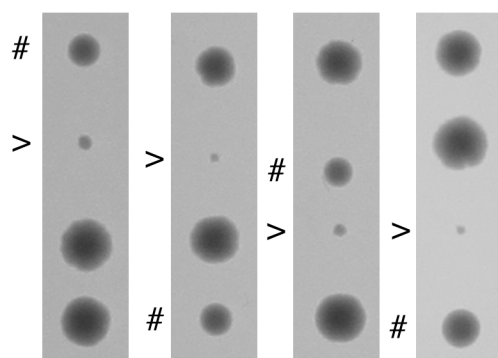

> *mip1Δ*  
# *mip1Δ gpa2Δ*

D

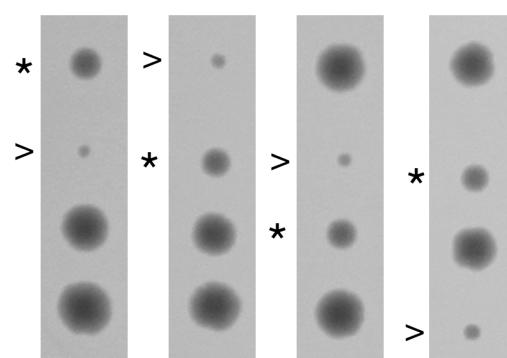

> *mip1Δ*  
\* *mip1Δ gpr1Δ*

Supplement: S3 Fig — (A) Sporulation-generated colonies depleted of mtDNA by Mip1p removal are larger when Pde2p may be overexpressed. Meiotic products of diploid strains CDD1020 (mip1Δ/MIP1 + 2μ-vector) and CDD1021 (mip1Δ/MIP1 + 2μ-PDE2) were dissected onto YEPD medium and incubated for 3 d. Images of mip1Δ colonies demonstrated to have inherited plasmid are provided at equivalent magnification. (B) Overexpression of Pde2p improves fitness of ρ0 cells generated by loss of the mtDNA polymerase. Haploid colonies originating from diploid strains CDD1022 (mip1Δ/MIP1 + 2μ-vector) or CDD1023 (mip1Δ/MIP1 + 2μ-PDE2) were verified for genotype, then struck from the SC-Ura test plate to SC-Ura medium and incubated at 30°C for 3 d. (C) Deletion of Gpa2p improves proliferation of cells made ρ0 by deletion of Mip1p. Diploid strain CDD1013 (gpa2Δ/GPA2 mip1Δ/MIP1) was sporulated, then spores were dissected onto YEPD medium and incubated for 3 d. (D) Deletion of Gpr1p speeds division of cells made ρ0 by removal of Mip1p. Diploid strain CDD1014 was treated as in (C). (PDF) [file pone.0146511.s003.pdf]

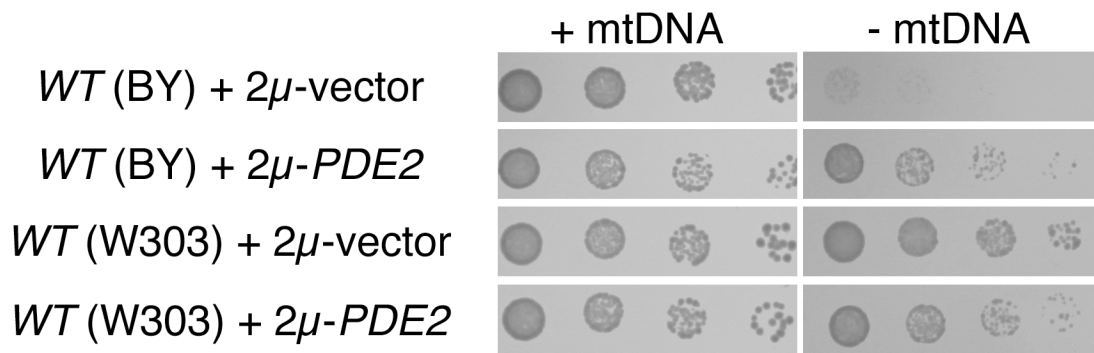

S4 Figure

Supplement: S4 Fig — Strains BY4741 (WT, BY background) and BMA64-1A (WT, W303 background) were treated as in Fig 1A. (PDF) [file pone.0146511.s004.pdf]

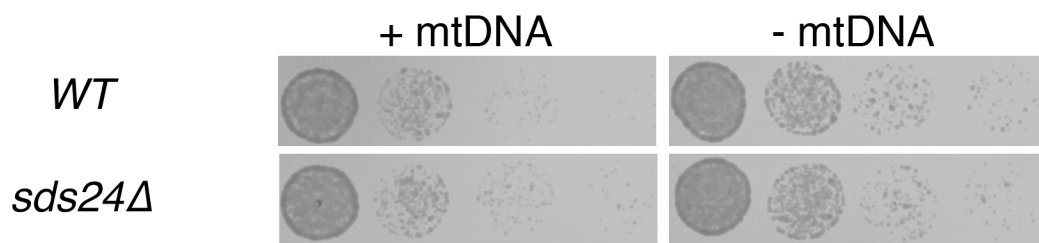

S5 Figure

Supplement: S5 Fig — Strains CDD463 (WT) and CDD912 (sds24Δ) were treated as in Fig 1B, except ρ+ cells were incubated on solid YEPD medium for 1 d, and ρ0 cells were incubated for 2 d. (PDF) [file pone.0146511.s005.pdf]

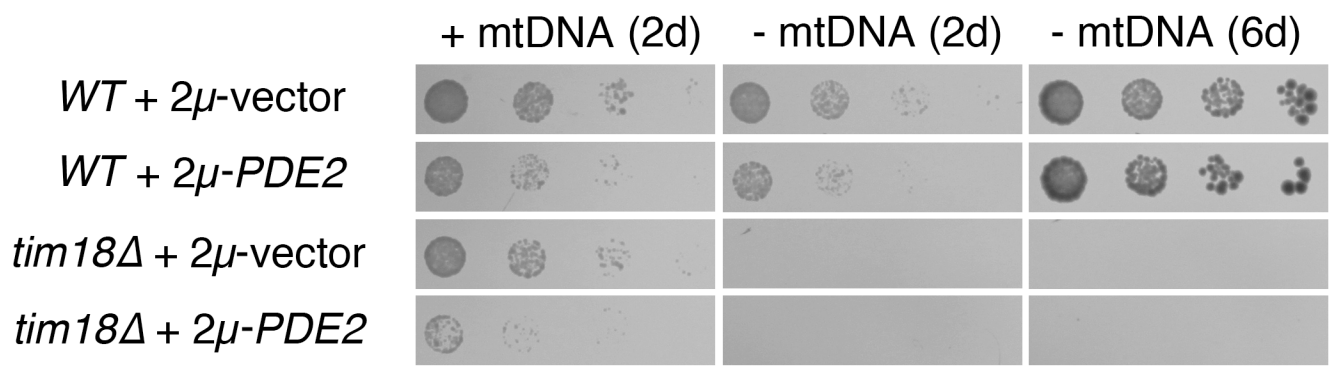

S6 Figure

Supplement: S6 Fig — Strains BY4741 (WT) and CDD9 (tim18Δ) were treated as in Fig 8C, except that cultures were proliferated and maintained at 37°C to circumvent the cold-sensitivity of the tim18Δ mutant. (PDF) [file pone.0146511.s006.pdf]

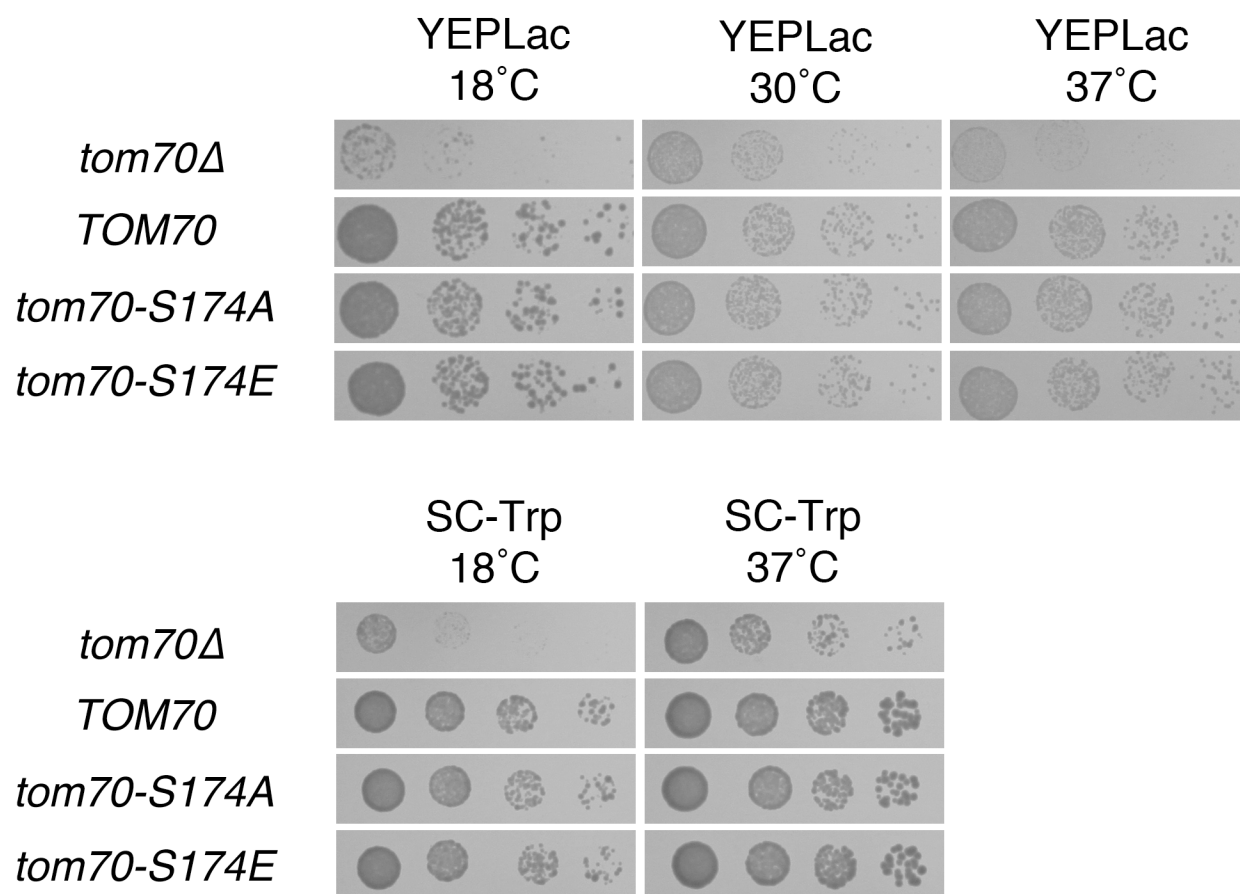

Supplement: S7 Fig — ρ+ cells transformants used in Fig 9 were also plated to the media indicated and incubated for 2 d (SC-Trp at 37°C, YEPLac at 30°C, YEPLac at 37°C); 4 d (SD-Trp at 18°C); or 7 d (YEPLac at 18°C). (PDF) [file pone.0146511.s007.pdf]

A

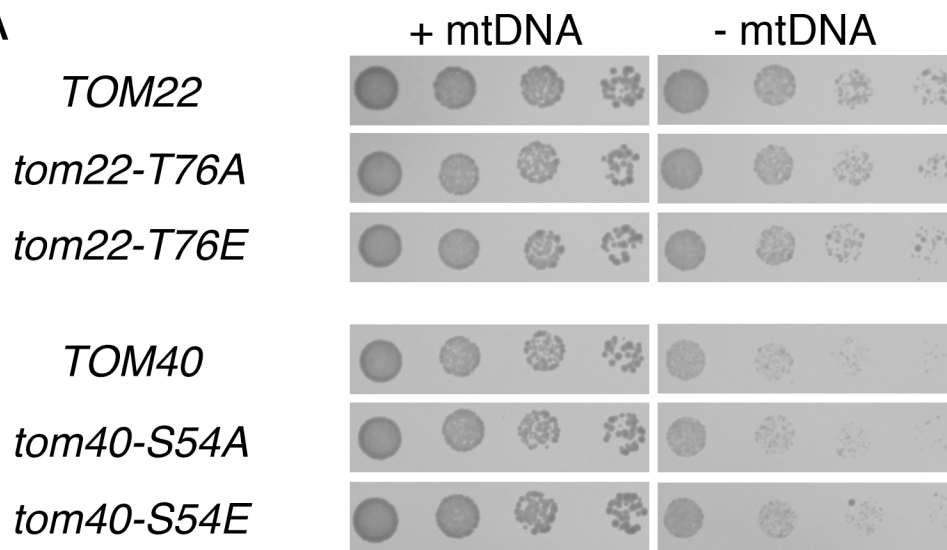

B

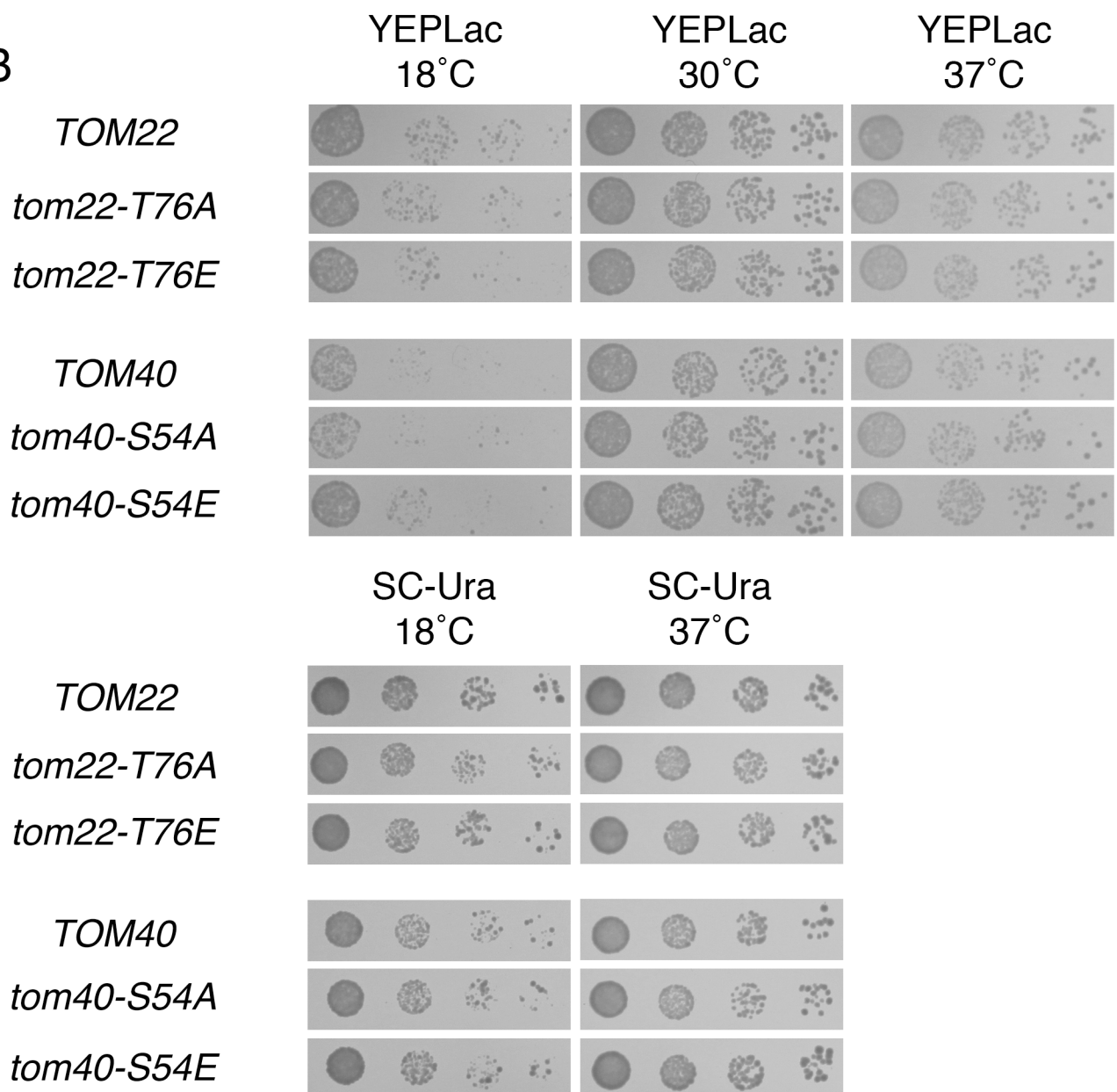

Supplement: S8 Fig — (A) Phosphorylation of T76 on Tom22p and phosphorylation of S54 of Tom40p do not determine the outcome of mtDNA damage. Strains CDD866 (TOM22), CDD867 (tom22-T76A), CDD868 (tom22-T76E), CDD869 (TOM40), CDD870 (tom40-S54A), and CDD871 (tom40-S54E), each manifesting chromosomal deletions complemented by plasmid-borne variants, were treated as in Fig 1A. Relevant genotypes are shown. (B) Phosphorylation of T76 on Tom22p and of S54 of Tom40p have no apparent consequence for cellular proliferation. ρ+ cultures used in (A) were plated to the media indicated and incubated for 2 d (SC-Ura at 37°C); 3 d (YEPLac at 30°C, YEPLac at 37°C), or 5 d (SC-Ura at 18°C, YEPLac at 18°C). (PDF) [file pone.0146511.s008.pdf]

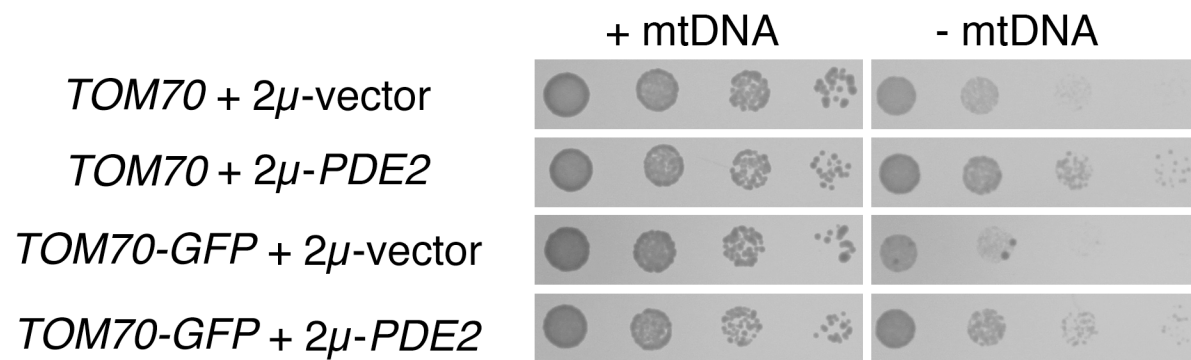

Supplement: S9 Fig — Strains CDD926 (TOM70-GFP) and CDD927 (TOM70) were treated as in Fig 1A. (PDF) [file pone.0146511.s009.pdf]
